# Deletion of the glucocorticoid receptor chaperone FKBP51 prevents glucocorticoid-induced skin atrophy

**DOI:** 10.18632/oncotarget.26194

**Published:** 2018-10-05

**Authors:** Gleb Baida, Pankaj Bhalla, Alexander Yemelyanov, Lance A. Stechschulte, Weinian Shou, Ben Readhead, Joel T. Dudley, Edwin R. Sánchez, Irina Budunova

**Affiliations:** ^1^ Department of Dermatology, Northwestern University, Chicago, IL, USA; ^2^ Department of Medicine, Pulmonary Division, Northwestern University, Chicago, IL, USA; ^3^ Department of Physiology & Pharmacology, The Center for Diabetes and Endocrine Research, University of Toledo College of Medicine, Toledo, OH, USA; ^4^ Wells Center for Pediatric Research, Indiana University School of Medicine, Indianapolis, IN, USA; ^5^ Department of Genetics and Genomic Sciences, Icahn School of Medicine at Mount Sinai, New York, NY, USA; ^6^ Institute for Next Generation Healthcare, Mount Sinai Health System, New York, NY, USA

**Keywords:** glucocorticoid, glucocorticoid receptor, skin atrophy, FKBP51, Akt

## Abstract

FKBP51 (FK506-binding protein 51) is a known co-chaperone and regulator of the glucocorticoid receptor (GR), which usually attenuates its activity. FKBP51 is one of the major GR target genes in skin, but its role in clinical effects of glucocorticoids is not known. Here, we used FKBP51 knockout (KO) mice to determine FKBP51's role in the major adverse effect of topical glucocorticoids, skin atrophy. Unexpectedly, we found that all skin compartments (epidermis, dermis, dermal adipose and CD34+ stem cells) in FKBP51 KO animals were much more resistant to glucocorticoid-induced hypoplasia. Furthermore, despite the absence of inhibitory FKBP51, the basal level of expression and glucocorticoid activation of GR target genes were not increased in FKBP51 KO skin or CRISPR/Cas9-edited FKBP51 KO HaCaT human keratinocytes. FKBP51 is known to negatively regulate Akt and mTOR. We found a significant increase in AktSer473 and mTORSer2448 phosphorylation and downstream pro-growth signaling in FKBP51-deficient keratinocytes *in vivo* and *in vitro*. As Akt/mTOR-GR crosstalk is usually negative in skin, our results suggest that Akt/mTOR activation could be responsible for the lack of increased GR function and resistance of FKBP51 KO mice to the steroid-induced skin atrophy.

## INTRODUCTION

Glucocorticoid hormones (Gcs) are steroid hormones mostly produced in the adrenal cortex. However, they can be also synthesized locally in different tissues including skin which expresses the genes for enzymes involved in glucocorticoid biosynthesis [1–3]. Gcs are well known physiological and pharmacological regulators of metabolism, proliferation, differentiation and inflammation in skin [4], and the decreased amount of endogenous Gcs in skin is now linked to the skin diseases such as psoriasis [5].

The synthetic Gcs were introduced as potent anti-inflammatory drugs in 1950-s, and remain the frontline for the treatment of inflammatory skin diseases, including psoriasis and atopic dermatitis [6, 7]. Unfortunately, chronic treatment with Gcs induces numerous adverse effects including cutaneous atrophy, which involves all skin compartments and results in overall skin thinning, reduction in size and number of keratinocytes, inhibition of dermal fibroblast proliferation and collagen synthesis, and atrophy of sebaceous glands, hair follicles and dermal adipose [8–10]. Although these morphological changes are well documented, the molecular mechanisms underlying development of skin atrophy are not well understood.

The effects of glucocorticoids are mediated by the glucocorticoid receptor (GR), a well-characterized transcription factor and member of the nuclear receptor family. In unstimulated cells, GR resides in cytoplasm bound to chaperones including heat shock proteins and immunophilins, such as FKBP51 (FK506-binding protein 51), which control GR activation and nuclear import [11]. Upon hormone binding, GR translocates to the nucleus where it regulates gene expression positively via binding to glucocorticoid response elements of target genes; or negatively via different mechanisms, including tethering – binding to other transcription factors, such as NF-κB, to modulate their activity [11]. In addition, GR can modulate in DNA-binding independent manner the pro-proliferative/pro-survival signaling mediated by phosphatidylinositol 3-kinase (PI3K), Akt, mTOR, and mitogen-activated protein kinases (MAPKs) [11–13].

We and others found that FKBP51 is a major GR target gene in skin, as it was strongly up-regulated in keratinocytes of skin-specific keratin5 (K5)-GR transgenics and down-regulated in GR knockout (KO) mice [14, 15]. FKBP51 is a member of FK506-binding protein (Fkbp) family. Like all immunophilins, FKBP51 has peptidyl-prolyl isomerase (PPIase) activity and is involved in immunoregulation, protein folding and trafficking [16]. As a molecular chaperone, FKBP51 has numerous clients including steroid receptors. In the case of GR, interaction with FKBP51 interferes with receptor maturation, reduces ligand-binding affinity and impairs GR nuclear import [17, 18]. Binding of ligand to GR favors replacement of FKBP51 with FKBP52, which then increases GR affinity to glucocorticoids, dimerization and nuclear transport [19].

FKBP51 is also known to control Akt, mTOR, and NF-κB pathways that in turn, negatively crosstalk with GR. Growth factor-dependent, full Akt activation requires phosphorylation at Thr308 and Ser473 [20]. FKBP51 acts as a scaffolding protein to enhance the interaction of the phosphatases PHLPP1/2 with Akt, which results in dephosphorylation of Ser473 [21, 22]. FKBP51 also binds to mTOR and inhibits its kinase activity as potently as rapamycin with its accessory partner FKBP12 [23]. In addition, FKBP51 impairs both NF-κB's nuclear translocation and transcriptional activity [24, 25]. The role of FKBP51 in the effects of topical glucocorticoids in skin has not been studied. Here, we confirmed FKBP51 induction by glucocorticoids in mouse and human skin, and used FKBP51 KO mice [26] to determine the role of FKBP51 in glucocorticoid-induced skin atrophy.

As FKBP51 is a major negative GR regulator, the possible outcomes in FKBP51 KO animals could be elevated GR activity and enhanced anti-proliferative effects of endogenous/exogenous glucocorticoids. However, we found that FKBP51 KO mice had modest epidermal hyperplasia and were much more resistant to skin atrophy induced by chronic treatment with the glucocorticoid fluocinolone acetonide (FA) than wild type mice. This effect in FKBP51 KO mice was seen despite the lack of changes in GR activity. We also found that Akt and mTOR were constitutively activated in FKBP51 KO skin and in HaCaT human keratinocytes lacking FKBP51. Taken as a whole, our results suggest that FKBP51 acts as an atrophogene during chronic skin treatment with steroids in part by inhibiting Akt/mTOR activity.

## RESULTS

### Topical glucocorticoids induce FKBP51 expression

FKBP51 is a GR target gene [14, 15]. However, the effect of glucocorticoids on FKBP51 in skin has not been well studied. We used a medium potency glucocorticoid fluocinolone acetonide (FA) to topically treat wild type F1 C57Bl x 129 mice, and a highly potent glucocorticoid clobetasol propionate (CBP) to treat human volunteers. The glucocorticoid doses and regimens used induce significant mouse and human skin atrophy, as shown previously [14, 27].

**Figure 1:**
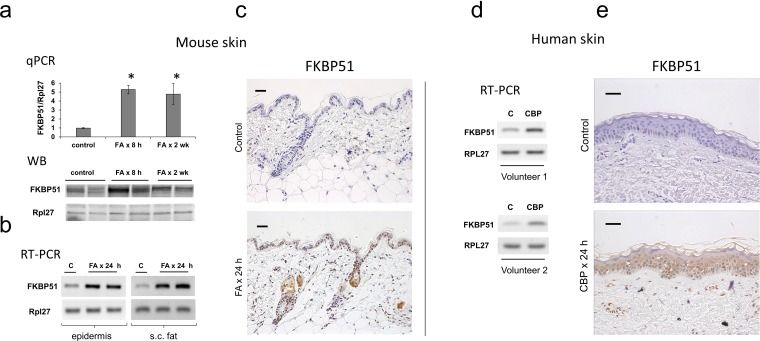
Induction of FKBP51 expression in the skin by glucocorticoids F1 C57Bl x 129 WT mice were treated topically with acetone (vehicle control, C) or glucocorticoid FA (2 μg/animal, for 8 h, 24 h or 2 wk). Skin of the inner upper arm of human volunteers was treated topically with 0.05% CPB cream once for 24 h; untreated skin from the opposite arm was used as control (C). FKBP51 expression was analyzed by qPCR and by Western blotting (WB) in murine epidermis **(a)**, by RT-PCR in epidermis and subcutaneous (s.c.) fat of mice **(b)**, or in whole thickness human skin biopsies **(d)**; Rpl27 was used as normalization/loading control. FKBP51 immunostaining of mouse **(c)** and human **(e)** skin. Scale bars are 20 μm **(c)** and 40 μm **(e)**. ^*^ P < 0.01, (unpaired two-tailed t-test) for changes compared to corresponding control.

**Figure 2:**
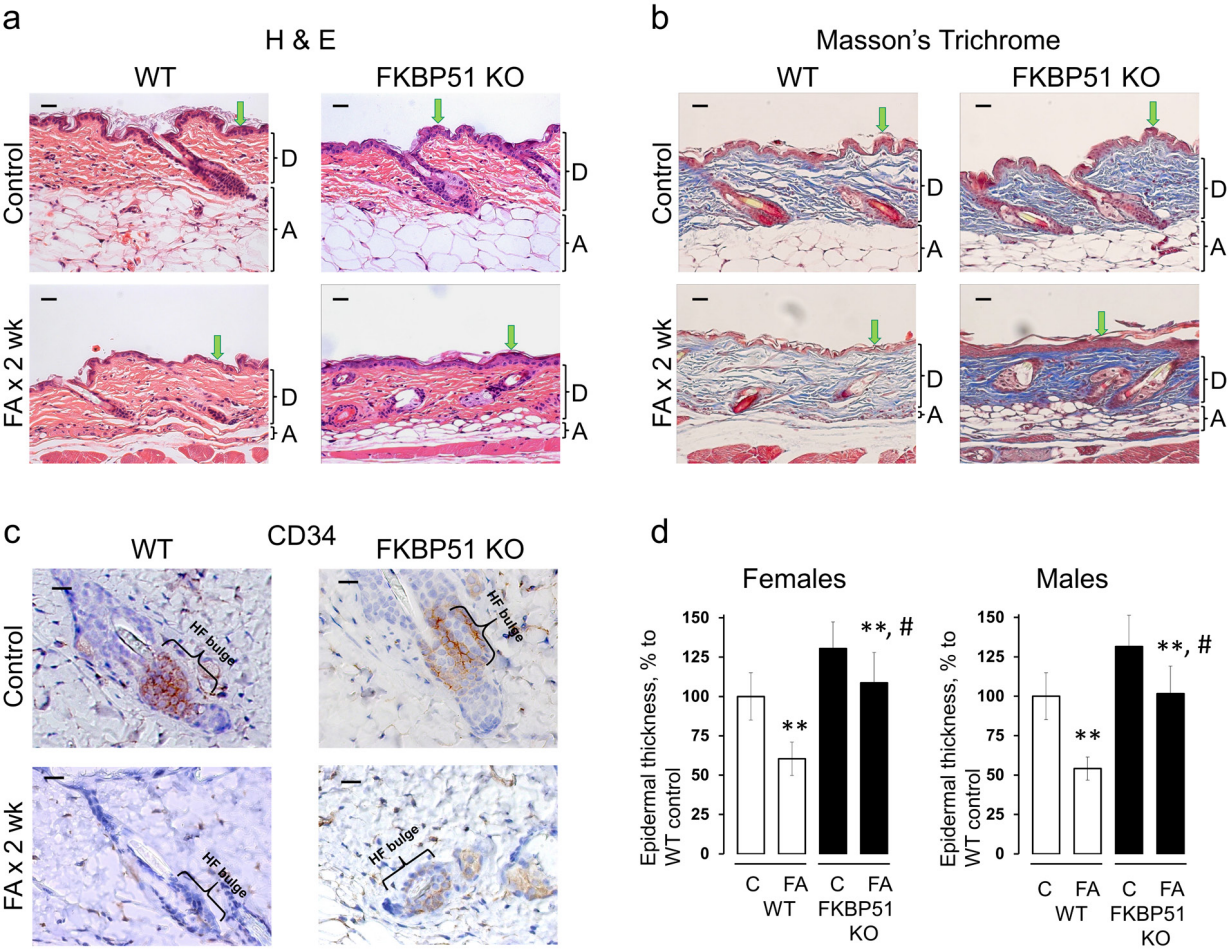
Resistance of FKBP51 KO mice to glucocorticoid-induced skin atrophy WT and FKBP51 KO male and female mice were treated with vehicle (Control) and FA (2 μg/animal) for 2 wks. **(a)** H&E; **(b)** Masson's trichrome staining; **(c)** CD34 immunostaining. Epidermis (green arrows), dermal adipose layer (A), dermis (D) and hair follicle bulge regions (HF bulge) are marked. Scale bars are 20 μm **(a, b)** and 10 μm **(c)**. **(d)** Quantitation of the epidermal thickness in female and male WT and FKBP51 KO mice was performed as described in Materials and Methods. Epidermal thickness is presented as % to corresponding control epidermis. The means ± SD were calculated for three individual skin samples in one representative experiment. ^**^ P < 0.001, (unpaired two-tailed t-test) for changes compared to corresponding control. ^#^ P < 0.001, (unpaired two-tailed t-test) for degree of reduction in epidermal thickness in FKBP51 KO animals compared to WT animals.

In mouse and human skin, the basal level of FKBP51 expression in epidermis and skin appendages was low (Figure 1). In mice, FKBP51 expression was strongly induced at mRNA and protein levels 8 h after FA application, and remained elevated during 2 wk treatment (Figure 1a-1c). FKBP51 protein was expressed in interfollicular epidermis, hair follicles and sebaceous glands in FA-treated skin (Figure 1c). Glucocorticoids-induced hypoplastic changes in mouse epidermis are always paralleled by the severe atrophy of dermal adipose [27], (Figure 2). Thus, we assessed FKBP51 expression in subcutaneous adipose mechanically separated from other skin tissues. The purity of adipose isolations was verified using specific adipocyte and keratinocyte markers (Supplementary Figure 1). We found strong, comparable to epidermis, induction of FKBP51 mRNA in adipose (Figure 1b).

To complement the animal study, we assessed the effect of glucocorticoid CBP on FKBP51 in human skin. We detected strong induction of FKBP51 mRNA and protein in human skin 24 h after CBP application (Figure 1d, 1e).

### Skin phenotype of FKBP51 KO mice

To determine the role of FKBP51 in glucocorticoid-induced skin atrophy, we used FKBP51 KO mice (Bl x 129 genetic background) that have no overt phenotype if untreated [26]. Interestingly, FKBP51 KO mouse skin was characterized by a modest, but significant, epidermal hyperplasia (Supplementary Figure 2a, 2c, 2d) and increased keratinocyte proliferation. In non-treated females the BrdU+ labeling indices were 4.2%+/−1.6 and 6.25%+/−2.5 in WT and FKBP51 KO mice accordingly (t-statistic, P<0.001). In males, there was also a trend to the increase in BrdU index in FKBP51 KO animals: 4.3% ± 1.5 in FKBP51 KO versus 2.7% ± 1.5 in WT epidermis (t-statistic, P=0.065). We also observed alteration of hair follicle morphology – unusual dilation of infundibulum frequently filled with flocculent mixture, especially in males (Supplementary Figure 2a, 2e). At the same time, there were no changes in the expression of keratinocyte differentiation markers K5, K10 and loricrin (Supplementary Figure 2b), suggesting that FKBP51 is involved in keratinocyte proliferation but not required for normal keratinocyte differentiation.

### Resistance of FKBP51 KO mice to glucocorticoid-induced skin atrophy

FKBP51 is a negative GR regulator, thus we expected that FKBP51 KO animals would be much more sensitive to the effects of glucocorticoids. Unexpectedly, FKBP51 KO mice appeared more resistant to skin atrophy than WT mice when subjected to FA chronic treatment. Epidermal thickness was reduced much less in both female and male KOs (by 16-22%) compared to WT mice (by 40-45%) (t-statistic, P<0.001; Figure 2a, 2d). In addition, dermal adipose was very strongly protected in the FKBP51 KO animals, especially in males. In females, the dermal adipose thickness was reduced at the end of two week FA treatment by 90% in WT compared to 60% in FKBP51 KO animals (Supplementary Figure 4). In males, the protective effect was even more pronounced: dermal adipose thickness was reduced in FA-treated WT skin by 80% while in FKBP51 KOs only by 10% (t-statistic P<0.001; Supplementary Figure 4). Interestingly, these results differed from our previous observations that FKBP51 positively controls adipogenesis *in vitro*, and in gonadal fat depot *in vivo* [28, 29].

Previously we found that GR activation sharply reduced the number and proliferative potential of epidermal stem cells (SCs) in the hair follicle bulge niche [14, 30]. Here, WT mice chronically treated with FA also showed almost a complete elimination of CD34^+^ follicular SCs (Figure 2c). Remarkably, in FKBP51 KO mice, the effect of FA on CD34 expression in the bulge was diminished, and ~ 25% of hair follicles remained CD34+ (Figure 2c), suggesting that the persistence of bulge SCs may have contributed to the protection of epidermis in KO mice against steroid atrophy.

The major atrophic effects of topical steroids in dermis include severe thinning of collagen and elastin fibrous networks and decreased dermal cellularity [10]. Masson's trichrome staining, which colors collagen blue, showed strongly reduced collagen fiber density in WT mice in contrast to FKBP51 KOs where effects on fiber network and dermal fibroblasts were minimal (Figure 2b).

It is known that genetic background can significantly affect phenotype of transgenic animals. Thus, we assessed the effect of FKBP51 KO on steroid skin atrophy using FKBP51 KO animals in C57Bl background. We found that FKBP51 KO provided even stronger protection against steroid-induced epidermal atrophy in C57Bl mice (Supplementary Figure 3).

### Effect of FKBP51 KO on GR activity and mTOR/Akt phosphorylation in skin

**Figure 3:**
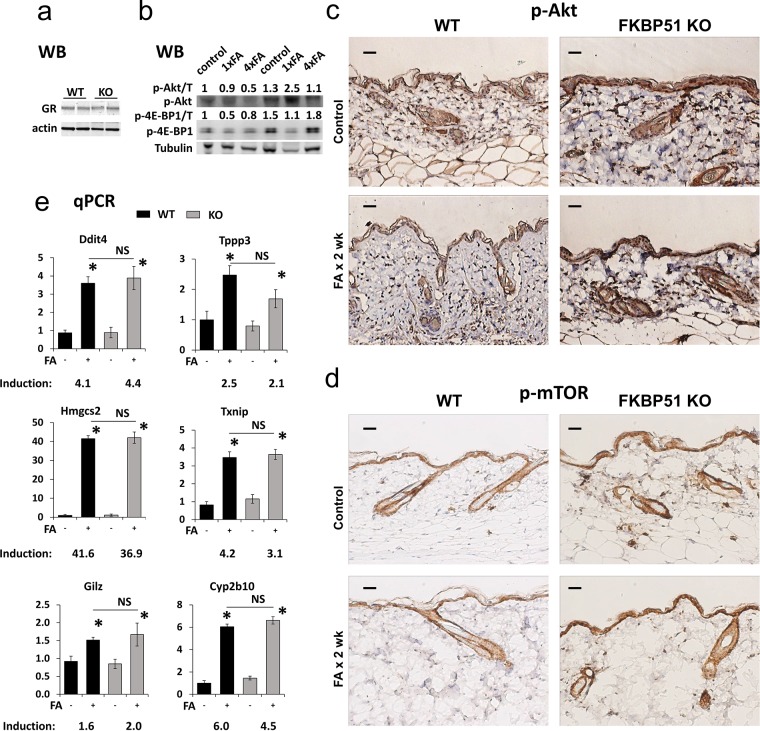
Effects of FKBP51 KO on GR function and Akt/mTOR phosphorylation in murine skin **(a)** Western blot (WB) analysis of GR expression in untreated C57Blx129 wild type (WT) and FKBP51 KO murine epidermis (two individual samples/condition). Actin was used as a loading control. **(b)** Western blot analysis of expression of phospho-Akt^Ser473^ and phospho-4E-BP1^Thr37/46^ in WT and FKBP51 KO murine epidermis treated with FA once for 24 h (1xFA) or four times during 2 weeks (4xFA) as described in Materials and Methods. Tubulin (T) was used as a loading control. ImageJ was used for densitometry. The intensity of p-Akt and p-4E-BP1 bands was normalized to the corresponding loading control (T) and expressed as fold of change vs vehicle-treated WT control. **(c, d)** Immunostaining for phospho-Akt^Ser473^ **(c)** and phospho-mTOR^Ser2448^ **(d)** in murine skin. Scale bars, 20 μm. **(e)** qPCR analyses of GR target genes expression in epidermis. Gene induction is fold change in expression after FA x 24 h (+) versus basal (−) expression in the same genotype. The results (normalized to Rpl27) are the means ± SD calculated for three individual RNA samples/condition. ^*^ P<0.05, (unpaired two-tailed t-test) for changes compared to corresponding control. NS - nonsignificant difference in FA-induction between WT and FKBP51 KO animals.

The reduced sensitivity of FKBP51 KO mice to glucocorticoid skin atrophy suggested that lack of FKBP51 could affect GR expression/signaling. We did not find significant changes in GR protein levels in epidermis of untreated FKBP51 KOs (Figure 3a). To assess changes in GR activity, we measured basal and FA-induced expression of known GR-target genes Tsc22d3/Gilz, Ddit4/Redd1, Cyp2b10, Txnip, Hmgcs2, and Tppp3, [27, 31–33], which were also revealed previously among the differentially expressed genes in skin of B6×129 WT mice treated with FA (our GEO Submission GSE59151). Surprisingly, despite the absence of inhibitory FKBP51, the glucocorticoid activation of these genes in KO mice was similar to that seen in WT epidermis (Figure 3e).

Other mechanisms by which FKBP51 could modulate GR signaling are through the inhibition of Akt, mTOR and/or NF-κB pathways [19, 20, 25], all of which negatively interact with GR signaling at different levels [11–13]. FKBP51 deficiency did not result in significant changes in the expression and phosphorylation of RelA/p65, the major NF-κB protein in mouse skin (data not shown). In contrast, we found strongly increased Akt^Ser473^ phosphorylation in the epidermis and appendages of vehicle- and FA-treated FKBP51 KO compared to WT mice, along with increased phosphorylation of mTOR^Ser2448^ (Figure 3c, 3d), the site targeted by Akt and mTOR effector ribosomal p70/S6 kinase1 (S6K1) [34–36]. These results are in agreement with our previous report on the Akt activation in FKBP51 KO murine fibroblasts [37]. We confirmed Akt and mTOR activation and their partial resistance to inhibitory effects of glucocorticoid FA in the skin of Fkbp51 KO animals using Western blot analysis (Figure 3b). Akt activity was assessed by Akt^Ser473^ phosphorylation, and mTOR activity - by phosphorylation of major mTOR downstream substrate 4E-BP1with easily detectable basal phosphorylation level in skin [38]. Surprisingly, we observed that in FKBP51 KO mice Akt^Ser473^ phosphorylation was increased by FA at earlier time-point of treatment.

### Effects of FKBP51 knockout on GR and Akt/mTOR function in human keratinocytes *in vitro*

**Figure 4:**
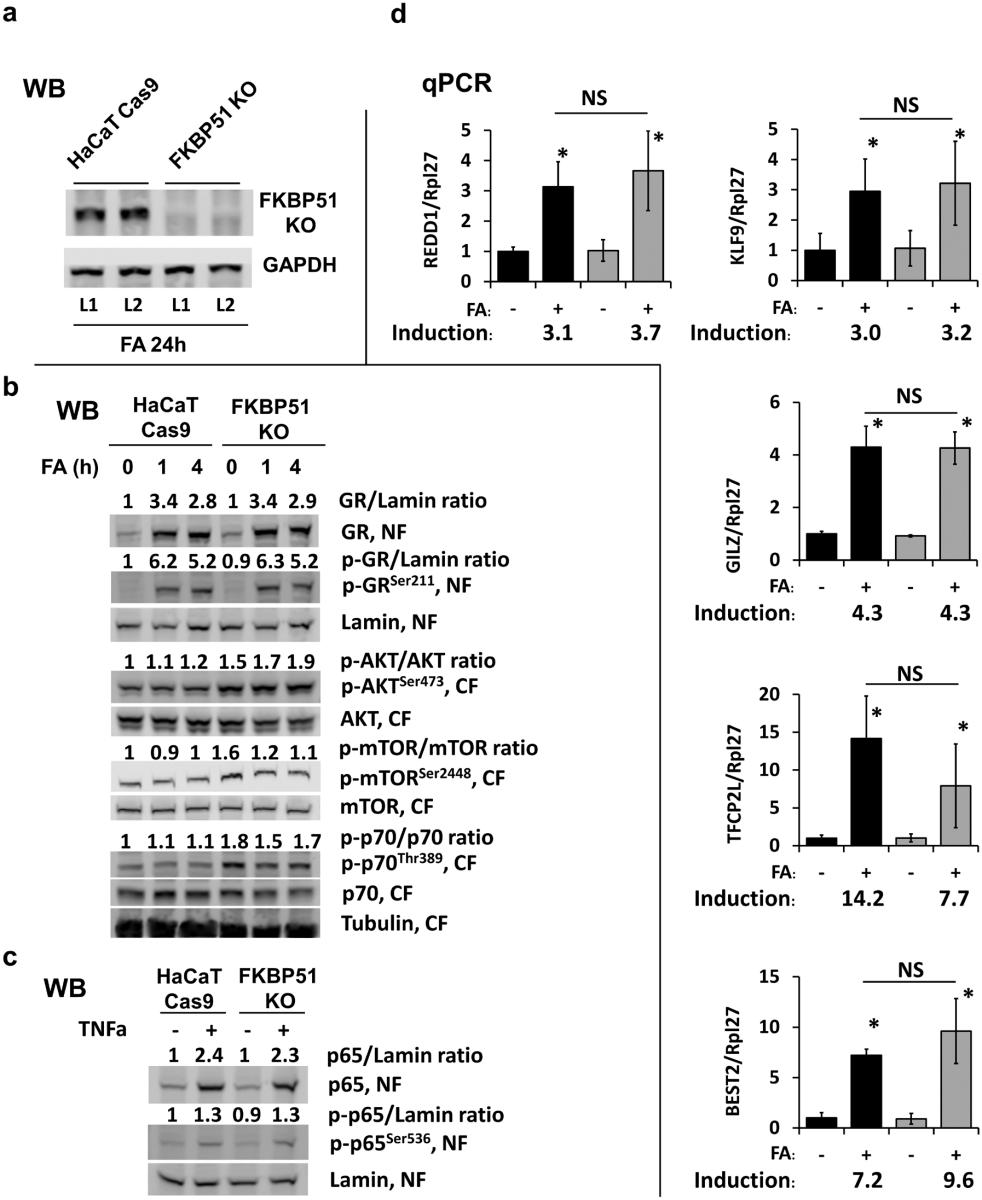
Effect of FKBP51 KO on glucocorticoid response and Akt/mTOR in HaCaT human keratinocytes FKBP51 KO and Cas9 (control) HaCaT human keratinocytes were treated with glucocorticoid FA (10^−6^ M) or vehicle as indicated. **(a)** Verification of FKBP51 knockout by Western blotting (WB), two independent FKBP51 KO clones (L1, L2) tested. GAPDH, loading control. **(b, c)** Western blot analyses for expression, phosphorylation, and subcellular localization of GR, Akt, mTOR, p70/S6K1, and NF-kB p65 in cells treated with FA (10^−6^ M x 0 (untreated), 1 h, and 4 h) **(b)**; or with TNF-a (50 μg/ml x 20 min) **(c)**. Nuclear (NF) and cytosol (CF) fractions were isolated as in Materials and Methods. Lamin and tubulin were used as nuclear and cytoplasmic loading controls, respectively. ImageJ was used for densitometry. For phosphorylated and total GR and p65, the intensity of bands was normalized to the corresponding loading controls and expressed as folds of change to vehicle-treated Cas9-HaCaT. For AKT, mTOR, and p70, the ratios of phosphorylation are expressed as folds of change to vehicle-treated Cas9-HaCaT. **(d)** qPCR analysis of GR target genes in cells treated with FA (10^−6^ M) for 24 h. The results (normalized to Rpl27) are the means ± SD calculated for three individual RNA samples/condition. Gene induction is fold of change in expression after FA (+) versus basal (−) expression. ^*^ P < 0.05 (unpaired two-tailed t-test) for changes compared to corresponding control. NS - nonsignificant difference in FA-induction between Cas9- and FKBP51 KO HaCaT cells.

To extend our findings and determine whether FKBP51 deficiency affects major steps of GR activation such as phosphorylation and nuclear import/retention, we generated FKBP51 KO keratinocytes using CRISPR/Cas9 technology. Nontumorigenic human HaCaT keratinocytes were transiently transfected with gRNAs in a complex with recombinant Cas9 to knockdown FKBP51 expression (FKBP51 KO-HaCaT). Control cells were transfected with Cas9 only (Figure 4a). Two FKBP51 KO-HaCaT cell lines were established from single cell clones, and used for experiments.

The results of Figure 4b show that loss of FKBP51 did not affect the amount of GR or its ability to translocate to the nucleus in response to glucocorticoid FA. Moreover, FA-induced GR phosphorylation at activating Ser211 and nuclear translocation/retention of phosphorylated GR were similar in FKBP51 KO-HaCaT and control HaCaT keratinocytes. We also did not find significant changes in activation of known GR target genes DDIT4/REDD1, KLF9, BEST2, TFCP2L1, and TSC22D3/GILZ [27, 30–32, 38] in FKBP51 KO keratinocytes (Figure 4d). These specific GR target genes were selected from the list of differentially expressed genes in HaCaT cells treated with FA (our GEO submission GSE97279, [38]).

In good correlation with *in vivo* results, we observed increased activation of Akt and mTOR after FKBP51 KO in HaCaT cells as assessed by phosphorylation of Akt^Ser473^, mTOR^Ser2448^, and mTOR effector ribosomal p70/S6 kinase1^Thr389^ (Figure 4b). At the same time, FKBP51 knockdown in HaCaT cells did not affect NF-kB function monitored by NF-kB protein RelA/p65 phosphorylation and nuclear translocation activated by TNF-a (Figure 4c).

## DISCUSSION

Glucocorticoid-induced skin atrophy represents a serious clinical problem as it leads to debilitating cosmetic effects and compromised skin barrier function [6]. The focus of this study was on the search for the molecular mechanisms underlying skin atrophy. We report here that the induction of FKBP51, a GR target gene involved in negative feedback loop in GR signaling, coincided with the development of cutaneous atrophy in humans and mice. Using FKBP51 KO mice, we discovered that FKBP51 acts as atrophogene, as in its absence all skin compartments and epidermal follicular SCs became protected from the hypoplastic effects of glucocorticoids.

The resistance of FKBP51 KO mice to steroid-induced skin atrophy was unexpected as FKBP51 is generally thought to be a negative regulator of GR activity [16, 17]. As a molecular chaperone that binds GR/Hsp90 heterocomplexes, FKBP51 has been shown to reduce GR affinity for ligands, to interfere with nuclear localization of receptor complexes under hormone-free conditions, and to alter GR phosphorylation [16, 18]. In light of these data, the lack of changes in GR phosphorylation at activating Ser211 in FKBP51 KO HaCaT keratinocytes was surprising. Equally unexpected was the lack of increased GR function in skin of FKBP51 KO mice and in keratinocytes after FKBP51 knockout suggesting that the concept of FKBP51 as a global negative regulator of GR should be revisited.

FKBP51 is a multi-functional protein: in addition to chaperoning steroid receptors, it negatively regulates activity of other signaling pathways such as Akt and mTOR, critical for protein synthesis and cell proliferation [19]. We report here that the levels of phospho-Akt^Ser473^, phospho-mTOR^Ser2448^ and phosphorylated downstream mTOR pro-proliferative effectors S6K1 and rpS6 were elevated in human keratinocytes lacking FKBP51 *in vitro* and in FKBP51 KO mouse skin *in vivo*.

The negative cross-talk between anabolic mTOR/Akt and catabolic GR signaling and inhibition of GR function by mTOR are well known in different tissues including muscle [13]. We recently showed that GR function is impaired in skin lacking mTOR/Akt inhibitor DDIT4/REDD1 [27]. The inhibitory effect of mTOR/Akt on GR could be mediated via direct phosphorylation of GR at Ser134, which negatively regulates GR activity [39]. Thus, increased activation of Akt/mTOR pro-metabolic/pro-proliferative signaling may help to explain the lack of GR activation as well as the protection of CD34^+^ stem cells, keratinocytes, dermal fibroblasts and adipocytes from glucocorticoids in FKBP51 KO mice.

Importantly, despite the increased activity of pro-proliferative Akt/mTOR signaling in the skin of FKBP51 KO mice, we observed only mild skin hyperplasia in these animals, and neither young nor aged FKBP51 KOs developed any cutaneous cancer lesions (data not shown). It is known that FKBP51 plays the important tissue-specific role in cancer development, and in skin it positively regulates melanoma stemness, growth, and metastatic potential [40–41]. However, its role in non-melanoma skin carcinogenesis remains to be investigated.

Interestingly, we identified recently another major target of glucocorticoids/GR, DDIT4/REDD1 (regulated in development and DNA damage response 1), as an atrophogene in skin [27, 38]. Like FKBP51, REDD1 is also a negative regulator of mTOR/Akt signaling [42, 43], and its genetic or pharmacological blockage spared skin from steroid-induced atrophy [27, 38]. Here we show that GR-mediated expression of REDD1 was unchanged in FKBP51 KO keratinocytes suggesting that down-regulation of FKBP51 alone is sufficient for partial resistance of skin to steroid-induced atrophy.

Overall, our studies highlight the complex, cell context-dependent regulation of GR function by FKBP51. Our results also imply that targeting of GR–dependent genes causatively involved in skin atrophy, such as FKBP51, could be a potential strategy to reduce/alleviate glucocorticoid-associated skin atrophy in the clinic.

Taking into consideration the potential anti-cancer effect of FKBP51 inhibition in skin [40] and similarity between atrophic processes in aged skin and skin chronically treated with glucocorticoids [44, 45], the same strategy could be relevant to a safe skin protection against aging. Importantly, FKBP51 belongs to the group of the genes whose expression in skin depends on age [46]. Since FKBP51 has been implicated in different neurological disorders, including post-traumatic stress disorder and Alzheimer's disease [18], efforts are already underway to develop FKBP51-specific inhibitors [47–48].

## MATERIALS AND METHODS

### Chemicals

FA and all other chemicals unless stated otherwise were purchased from Sigma (St. Louis, MO). CBP (0.05% cream) was purchased at the pharmacy.

### Animals and treatments

B6×129 (F1 C57BL/6 × 129SvEv) and C57Bl/6 wild-type (WT) mice were from Taconic (Germantown, NY). FKBP51 KO mice on B6×129 genetic background were generated by an insertional mutation in intron 4 of the Fbkp5 gene [26]. Later transgene was transferred into C57Bl/6 genetic background. Seven-wk old mice were shaved and treated 3 days later. FA was applied topically (2 μg/200 μl acetone) to the back skin once or 4 times every third day for two weeks as described [14]. Control animals were treated with acetone only. Skin was harvested 4-24 h after the last application as indicated in the Figure legends. Mice were injected ip with BrdU (50 μg/g body weight) 1 h before sacrifice. For biochemical studies, epidermis and subcutaneous fat were mechanically isolated from the skin by scraping [14].

All animal experiments were approved by the Northwestern University Animal Care and Use Committee.

### Human volunteers

CBP cream (0.05%) was applied topically to the inner skin of the upper right arm of healthy human volunteers (age 32-45) once. Untreated left arm skin was used as control. Five mm punch skin biopsies were taken 24 h after CBP application and processed for RNA extraction or formalin-fixed for immunohistochemistry. All human studies were approved by Northwestern University Institutional Review Board. Written informed consent was received from the volunteers before participation.

### Human keratinocyte cell cultures

HaCaT human keratinocyte cells are *in vitro* spontaneously-immortalized keratinocytes from histologically normal human skin [49]. Cells were kindly provided by Dr. K. Green (Northwestern University, Chicago). HaCaT cells were cultured in Dulbecco's Modified Eagle medium containing 10% FBS (Cellgro; Manassas, VA) and antibiotics.

### Knockout of FKBP51 expression by CRISPR/Cas9 gene-editing

CRISPR/Cas9 gene-editing was used to ablate FKBP51 in HaCaT cells. Recombinant Cas9 protein and CRISPR crRNA were purchased from Integrated DNA Technologies (Coralville, IA). The gRNAs were designed using publicly accessible engines (http://crispr.mit.edu/; http://chopchop.cbu.uib.no/ (http://www.uib.no/en/persons/) as described [50–52]. Only top ranked gRNA with no off-target effects confirmed by two design sites were selected for the knock-out procedure (gRNA1, FKBP51 exon 2, ATTACCTCCAAAAAAGACAG; gRNA2, FKBP51 Exon 3, GGTGAGGAAACGCCGATGAT; gRNA3, FKBP51 Exon 4, GTCATCAAGGCATGGGACAT). HaCaT cells were transiently transfected with ribonucleoprotein complex (RNPC) using RNAi-Max transfection reagent (Fisher Scientific). The advantages of RNPC (with Cas9 protein and synthetic gRNAs) versus standard vector-based CRISPR approaches include rapid RNPC degradation after gene editing resulting in minimal Cas9-gRNA off-target effects and cytotoxicity. Single-cell colonies were established from CRISPR-edited cell cultures and analyzed by sequencing and Western blotting to verify the knock-out of FKBP51 gene. Cas9-only-treated cells were used as a control. Experiments were performed in two different FKBP51 KO clones (L1 and L2).

### Histological analysis and immunostaining

Formalin-fixed, paraffin-embedded skin sections were stained with hematoxylin and eosin (H&E) and/or antibodies against BrdU (BD Biosciences; San Jose, CA), keratinocyte differentiation markers keratins 5 and 10 and loricrin (Covance; Greenfield, IN), FKBP51 and GR (Santa Cruz; Dallas, TX), phospho-mTORSer^2448^, phospho-Akt^Ser473^ (Cell Signaling; Danvers, MA), stem cell marker CD34 (Abcam; Cambridge, MA) and Masson's trichrome to assess dermal collagen density/organization.

Quantification of the epidermal and dermal adipose thicknesses (as the readouts for skin thinning) was performed in H&E stained skin sections. At least 10 individual fields/slide in three individual samples/experimental group were counted using Axioplan2 microscope software (Carl Zeiss). To assess keratinocyte proliferation, the number of proliferating (BrdU^+^) and total basal keratinocytes was counted in 10 individual fields/slide of each sample.

To assess the effect of FA on the expression of CD34 stem cell marker, we examined ~ 100 hair follicles in the skin samples of each genotype (3 individual skin samples/group). Hair follicles with ≥5 CD34+ cells in the bulge, were considered positive.

### Western blot analysis

Whole cell protein extracts were prepared as described [27]. Separate cytoplasmic and nuclear protein fractions were isolated with NE-PER Nuclear and Cytoplasmic Extraction Kit according to manufacturer protocol (ThermoFisher Scientific, Waltham, MA). The proteins were resolved by SDS-PAGE and transferred to nitrocellulose membranes (LI-COR Biosciences; Lincoln, NE). After blocking, membranes were incubated with primary antibodies overnight at 4°C, followed by IRDye® secondary antibodies (LI-COR Biosciences). LI-COR Odyssey Imager was used for the band visualization. The antibodies against FKBP51, GR, RelA/p65, p65^Ser536^ (Santa Cruz Biotechnology, Dallas, TX), Redd1 (Proteintech; Chicago, IL), phospho-GR^Ser211^, Akt, phospho-Akt^Ser473^, phospho-rpS6^Ser240/244^, phospho-p70/S6K^Thr389^, lamin B, tubulin (Cell Signaling, Danvers, MA), and GAPDH (Sigma, St Louis, MO) were used at concentrations recommended by their manufacturers. The multi-band pattern of FKBP51 signal on Western blots (Figure 1a) may reflect FKBP51 post-translational modifications such as phosphorylation [41].

ImageJ (http://rsb.info.nih.gov/ij/index.html) was used for densitometry. The intensity of bands was normalized to the corresponding loading control and expressed as fold of change vs vehicle-treated WT or Cas9-only control.

### RNA preparation and PCR

RNA from whole human skin, murine epidermis and keratinocyte cultures were isolated with RiboPure kit (Ambion/Life Technologies; Grand Island, NY), and from subcutaneous adipose with RNeasy Lipid Tissue Kit (Qiagen; Germantown, MD) and treated with TURBO™ DNase (Ambion). Purity of RNA samples from epidermis and subcutaneous fat was confirmed by keratinocyte and adipose cell markers expression. Gene expression was assessed using semi-quantitative two-step RT-PCR and quantitative real-time PCR (qPCR). Reverse transcription was performed using 1 μg RNA, random hexamers and M-MLV reverse transcriptase (Invitrogen/Life Technologies), according to manufacturer instructions as described [27]. The gene-specific primers were designed with NCBI Primer-BLAST as described [27, 38]. qPCR with SYBR Green detection was performed on the LightCycler® 96 Real-Time PCR instrument (Roche Diagnostics, Indianapolis, IN). Each sample was tested in triplicate. The results were normalized to the expression of the housekeeping Rpl27 gene [53].

### Statistical analysis

Mean and standard deviation (SD) values were calculated using Microsoft Excel software. The treatment effects in each experiment were compared by t-test using the GraphPad (La Jolla, CA) statistical software package. Differences between groups were considered significant at P < 0.05. All experiments were repeated 2-3 times. In animal experiments we used 3-4 mice per experimental group. In all figures, the results of one representative experiment are shown as means ± SD.

## SUPPLEMENTARY MATERIALS FIGURES



## Abbreviations

BrdU: 5-bromo-2′-deoxyuridine
CBP: clobetasol propionate
CRISPR: Clustered Regularly Interspaced Short Palindromic Repeats
FA: fluocinolone acetonide
FKBP51: FK506-binding protein 51
GAPDH: glyceraldehyde 3-phosphate dehyddrogenase
GR: glucocorticoid receptor
K5: keratin-5
K10: keratin-10
KO: knockout
MAPK: mitogen-activated protein kinase
mTOR: mammalian target of rapamycin
NF-κB: nuclear factor-kB
PDK1: 3-phosphoinositide-dependent protein kinase 1
PHLPP: pleckstrin homology domain leucine-rich repeat protein phosphatase
PI3K: phosphatidylinositol 3-kinase
PPIase: *cis-trans* peptidyl-prolyl isomerase
qPCR: quantitative real-time reverse-transcribed polymerase chain reaction
Rpl27: ribosomal protein L27
rpS6: ribosomal protein S6
S6K1: ribosomal p70/S6 kinase 1
SC: stem cell
WT: wild-type
