# Supplementary material for: Deletion of the glucocorticoid receptor chaperone FKBP51 prevents glucocorticoid-induced skin atrophy
Source: Oncotarget. 2018 Oct 5;9(78):34772–83. doi: 10.18632/oncotarget.26194 (PMC6205168; doi:10.18632/oncotarget.26194)
Supplement: Supplementary file 1 [file oncotarget-09-34772-s001.pdf]

Deletion of the glucocorticoid receptor chaperone FKBP51 prevents glucocorticoid-induced skin atrophy

SUPPLEMENTARY MATERIALS

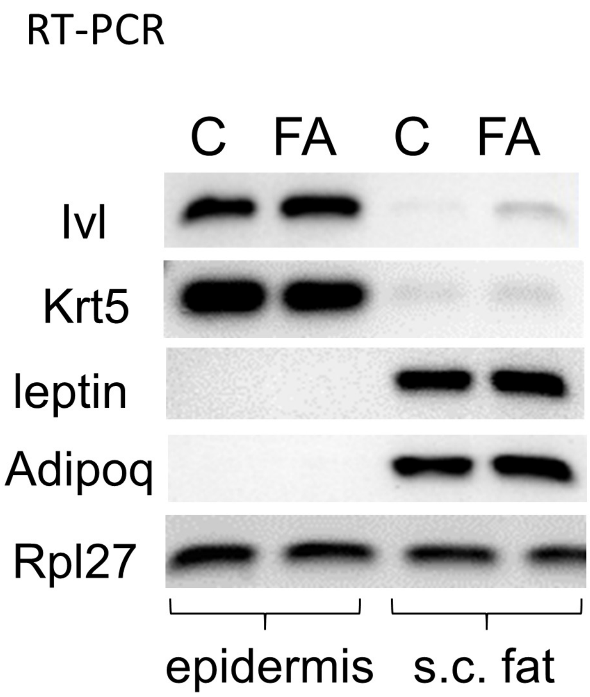

Supplementary Figure 1: Adipocyte and keratinocyte marker expression in epidermis and subcutaneous adipose RNA extracts. Total RNA from epidermis and subcutaneous (s.c.) adipose was isolated and subjected to RT-PCR as described in Materials and Methods. Epidermal and adipose RNA purity was confirmed by evaluation of expression of epidermal (keratin5 [Krt5] and involucrin [Ivl]) and adipose (leptin and adiponectin [Adipoq]) markers in epidermis and s.c. fat from control (C) B6 x 129 mice and mice treated with FA for 24 h (FA). Rpl27 was used as a cDNA loading control.

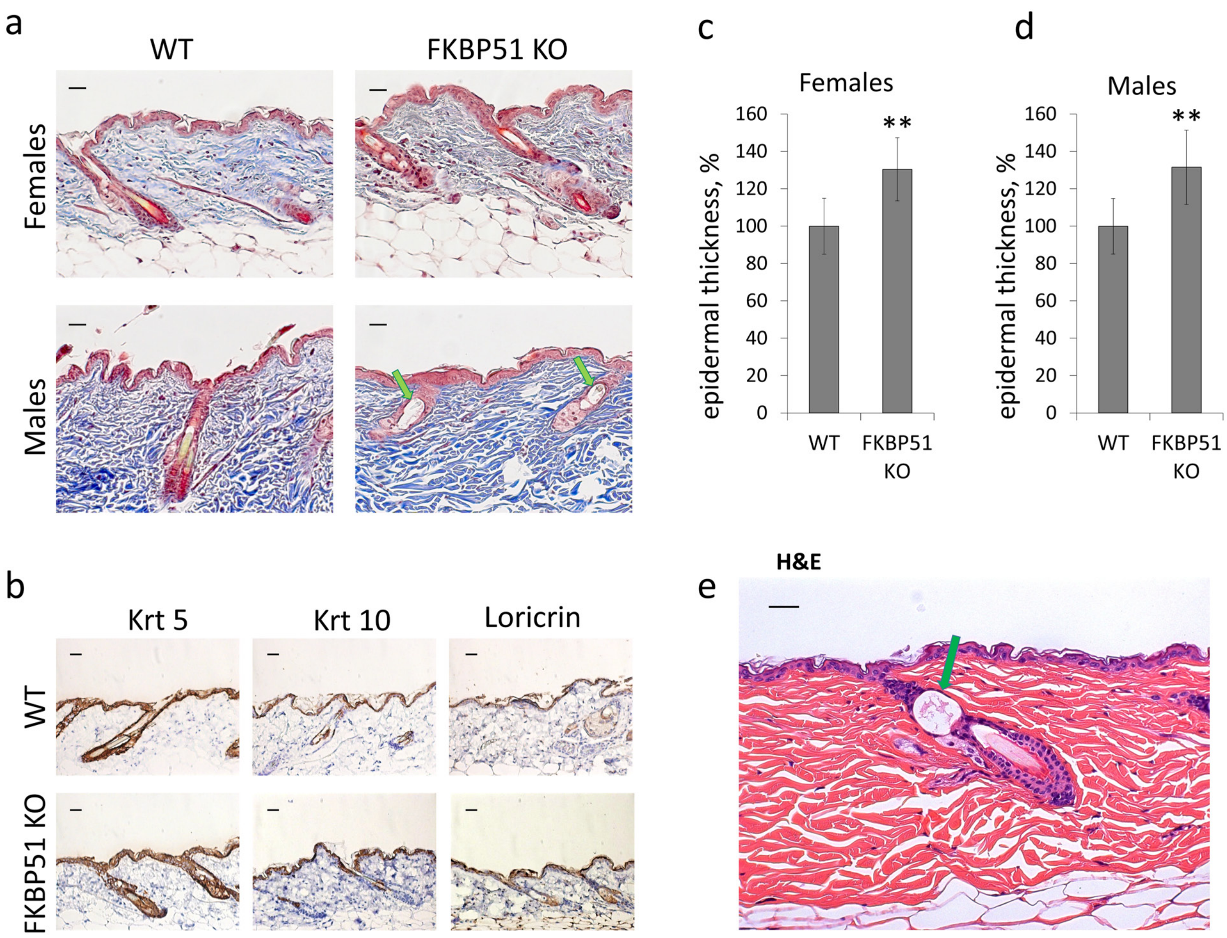

Supplementary Figure 2: Hyperplastic skin phenotype and morphological changes in hair follicles in FKBP51 KO untreated animals. **(a)** Masson's tri-chrome staining of B6x129 WT and FKBP51 KO female and male skin. Green arrows: dilated infundibulum in hair follicles. **(b)** Immunochemical staining of untreated B6x129 WT and FKBP51 KO murine skin for expression of epidermal keratinocyte markers keratin 5, keratin 10, and loricrin. Scale bars are 20 μm. **(c, d)** Morphometric analysis of the sections of B6x129 WT and FKBP51 KO female **(c)** and male **(d)** mouse skin. FKBP51 KO epidermal thickness is presented as % to thickness of WT epidermis. Statistical analysis for differences between WT and FKBP51 KO animals was done by unpaired two-tailed t-test. **P < 0.01. **(e)** Morphological changes in hair follicles in male skin – dilated infundibulum often filled with flocculent material (green arrow). Scale bar is 20 μm.

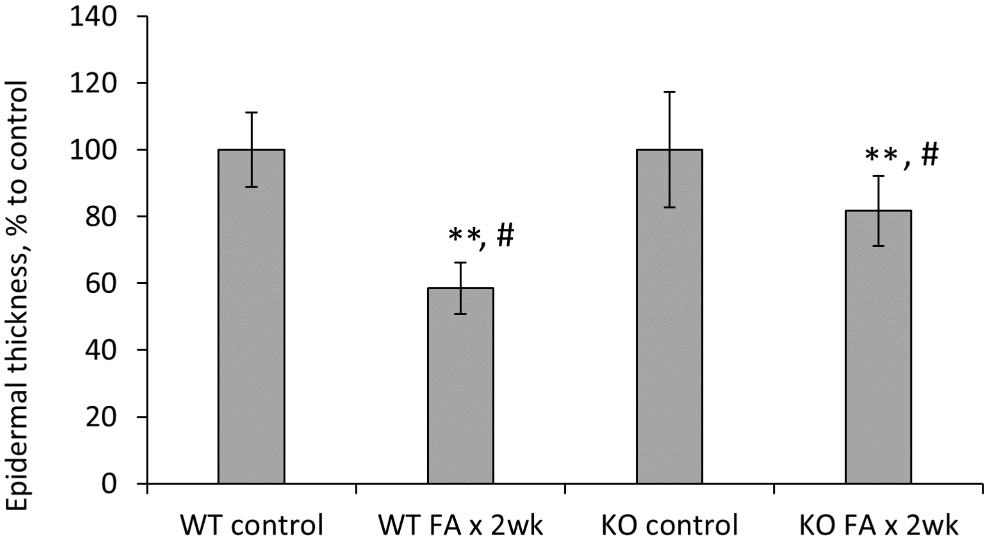

Supplementary Figure 3: Resistance of FKBP51 KO mice in C57Bl genetic background to glucocorticoid-induced skin atrophy. Morphometric analysis of epidermal thickness in C57Bl WT and FKBP51 KO female mice treated with vehicle control or FA (2 µg/animal) every 72 h for 2 wks. Quantitation of the epidermal thickness in female WT and FKBP51 KO mice was performed as described in Materials and Methods. Epidermal thickness is presented as % to corresponding control epidermis. The means ± SD were calculated for three individual skin samples in one representative experiment. ** $P < 0.001$ (unpaired two-tailed t-test) for changes compared to corresponding control. # $P < 0.001$, (unpaired two-tailed t-test) for degree of reduction in epidermal thickness in FKBP51 KO animals compared to WT animals.

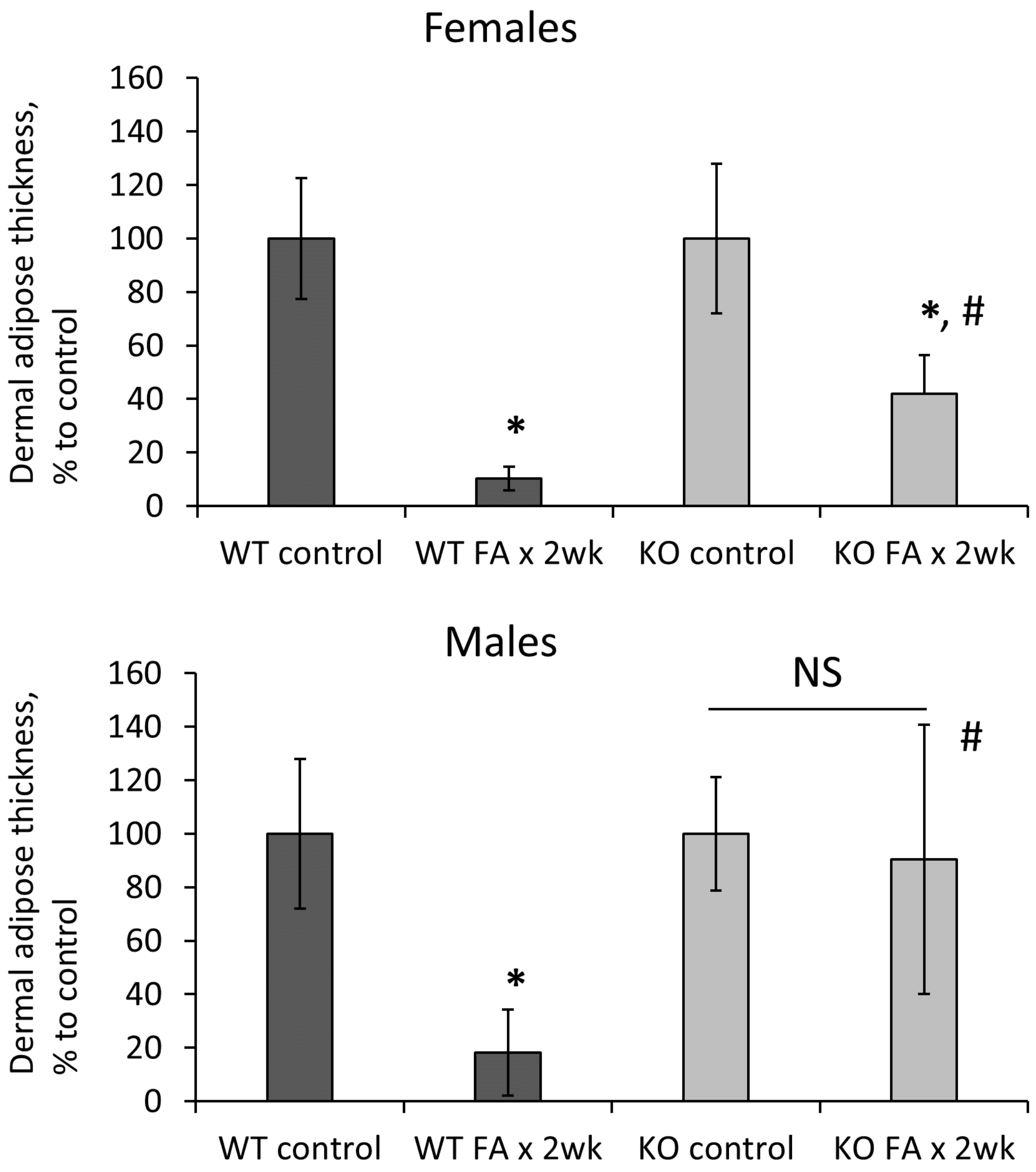

Supplementary Figure 4: Resistance of dermal adipose in FKBP51 KO mice to glucocorticoid-induced atrophy. Morphometric analysis of dermal adipose thickness in C57Bl WT and FKBP51 KO female and male mice treated with vehicle (control) or FA (2 µg/animal) every 72 h for 2 wks. Quantitation of the dermal adipose thickness was performed as described in Materials and Methods. Statistical analysis for differences between the groups was done by unpaired two-tailed t-test. * $P < 0.0001$ (unpaired two-tailed t-test) for changes compared to corresponding control. NS - nonsignificant difference. # $P < 0.001$, (unpaired two-tailed t-test) for degree of reduction in dermal adipose thickness in FKBP51 KO animals compared to WT animals of the same sex.
